# Genetic profiling and precision skin care: a review

**DOI:** 10.3389/fgene.2025.1559510

**Published:** 2025-06-03

**Authors:** Barbara Geusens, Diala Haykal

**Affiliations:** ^1^ Nomige, Gent, Belgium; ^2^ Private Practice, Centre Médical Laser Palaiseau, Paris, France

**Keywords:** dermagenetics, genetic profiling, multi-omics, precision dermatology, skin aging, polygenic risk scores, epigenetics

## Abstract

Skin aging is a multifaceted biological phenomenon driven by intrinsic and extrinsic factors, including genetics, hormonal changes, metabolic shifts, and environmental influences. Notably, genetic factors play a significant role, explaining up to 60% of the variability in how individuals age. Genes such as elastin (ELN), filaggrin (FLG), and melanocortin 1 receptor (MC1R) play pivotal roles in processes like elasticity, hydration, and pigmentation, directly impacting both intrinsic and extrinsic aging pathways. Understanding these genetic mechanisms is crucial for advancing personalized anti-aging products and therapies, particularly given the significant variability among individuals and ethnic groups. This review explores the current state of knowledge regarding the genetic determinants of skin aging, highlighting recent discoveries and proposing functional pathways for targeted interventions. Future directions are discussed to highlight the transformative potential of these innovations in clinical and aesthetic dermatology. While genetic factors may account for up to 60% of skin aging variability in specific populations, this figure should be interpreted with caution. It primarily reflects heritability under controlled conditions and does not negate the significant influence of modifiable lifestyle and environmental factors on skin and overall aging.

## 1 Introduction

Skin aging is a complex process influenced by intrinsic factors like genetics and metabolism, and extrinsic factors such as UV radiation, lifestyle and pollution (Farage et al., 2008; Kim and Park, 2016). These factors contribute to structural and functional changes in the skin, including loss of elasticity, increased dryness, and pigmentation alterations (Lee et al., 2021). At the molecular level, aging skin experiences collagen decrease, elastin degradation, and oxidative stress-induced damage (Uitto, 2008; Naidoo and Birch-Machin, 2017). Cellular senescence and the accumulation of senescent cells play a significant role in skin aging, leading to the degradation of the extracellular matrix (Shin et al., 2023). The aging process affects both the epidermis and dermis, with histological changes such as epidermal atrophy and decreased fibroblast numbers (Lee et al., 2021; Zhang and Duan, 2018). Understanding these mechanisms is crucial for developing effective anti-aging treatments and improving the quality of life for the aging population (Farage et al., 2007).

The ability to study the main contributors to skin health, including genetics, diet, and environmental exposures, has introduced the concept of personalized skincare. By analyzing the genetic background of an individual, it becomes possible to identify specific skin characteristics, assess risks for developing certain skin concerns, and recommend precision treatments or skincare regimens tailored to individual needs. For example, individuals with genetic markers for premature collagen degradation or heightened oxidative stress sensitivity can access targeted interventions to preemptively address these risks (Geusens et al., 2020; Khorsandi et al., 2016).

This approach moves beyond the traditional one-size-fits-all model, empowering individuals to take proactive control of their skin health. Moreover, personalized skincare minimizes the frustration often associated with ineffective products by replacing the trial-and-error process with solutions grounded in evidence-based science. Patients can see measurable improvements, which fosters trust in prescribed regimens and enhances their overall satisfaction.

Beyond physical outcomes, this approach addresses the emotional dimensions of skincare. Visible improvements in skin health often translate into heightened self-esteem, as individuals feel empowered by treatments aligned with their unique biology. Personalized skincare also encourages broader lifestyle changes by integrating genetic insights with advice on nutrition, stress management, and environmental exposures, creating a comprehensive framework for long-term skin health and overall wellbeing. This holistic approach underscores the interconnected nature of external appearance and internal health, promoting mindfulness and sustained care.

This narrative review was conducted by identifying relevant literature through systematic searches in PubMed, Scopus, and Google Scholar. Keywords included “skin aging,” “genetics,” “single nucleotide polymorphisms (SNPs),” “personalized skincare,” “epigenetics,” “polygenic risk score (PRS),” “precision dermatology,” “microbiome,” and “multi-omics.” Studies published between 2000 and 2024 were prioritized, focusing on peer-reviewed articles and clinical evidence related to genetic, epigenetic, and environmental influences on skin aging. The selection criteria emphasized mechanistic insights, human clinical relevance, and implications for personalization. Reference inclusion was guided by relevance to functional pathways and potential translational application.

## 2 The growing trend of personalization in medicine and dermatology

Personalization has become a defining trend across numerous fields, particularly in medicine and dermatology. Advances in technology and research have enabled a shift from generic, one-size-fits-all approaches to treatments tailored to the unique characteristics of each individual.

The field of personalized medicine focuses on understanding genetic profiles to target specific biological pathways, enhancing the precision and effectiveness of treatments. Pharmacogenomics, a cornerstone of personalized medicine, examines how genetic variations influence individual drug responses, aiming to optimize therapy and minimize adverse effects (Althagafi et al., 2022; Dash et al., 2024). This field has the potential to revolutionize healthcare by tailoring treatments based on patients’ genetic profiles (Mini and Nobili, 2009; Topić, 2008). Pharmacogenetic biomarkers, both prognostic and predictive, are crucial in classifying patients into subgroups for treatment recommendations (Chen et al., 2013). While pharmacogenomics has shown promise in various therapeutic areas, including cancer, diabetes, and cardiovascular diseases, its implementation in clinical practice has been slow due to the complexity of drug actions and genetic variations (Sadee and Dai, 2005; Ramayanam et al., 2022).

Personalized medicine in dermatology has gained momentum, leveraging genetic insights to develop targeted therapies for various skin conditions (Schweitzer and Maibach, 2014; Rizzo and Maibach, 2011). Biomarkers play a crucial role in this personalized approach, aiding in diagnosis, prognosis, and therapeutic monitoring for conditions such as psoriasis, atopic dermatitis, and skin cancers (Tan et al., 2024; Brownstone et al., 2021). Genomic libraries and genetic markers enable dermatologists to identify relevant genetic alterations and abnormal signaling mechanisms, particularly in melanoma, allowing for more effective treatment selection (Griewank et al., 2014; Nathanson, 2010). The integration of molecular profiling and sophisticated informatics platforms has led to the development of dermatologic disease-directed targeted therapies (D3T2), improving patient outcomes and reducing healthcare costs (Cohen and Kurzrock, 2022).

In atopic dermatitis, serum TARC/CCL17 is the most promising biomarker for assessing disease severity (Thijs et al., 2015; Mastraftsi et al., 2022). For psoriasis, biomarkers like TNF-α and IL-23 guide targeted therapies (Tan et al., 2024). In melanoma, biomarker-driven treatment paradigms have become common (Brownstone et al., 2021). Currently, six dermatological drugs have recommended or mandatory biomarker testing, mostly for melanoma and HIV-related conditions (Landeck et al., 2016). Novel techniques like tape strip profiling and microneedle-based patches are being developed for non-invasive biomarker collection (Mortlock et al., 2023). While numerous potential biomarkers have been identified, only a few have achieved full validation for clinical applications (Bakker et al., 2023). This underscores the need for continued research to bridge this gap. The integration of pharmacogenomics and biomarkers in dermatology is advancing, with the FDA approving biomarkers for certain drugs (Do and Maibach, 2019).

## 3 Personalized skincare and ethnic diversity

Personalized skincare, although distinct from medical dermatology, serves as a complementary approach by focusing on cosmetic improvements and preventive care tailored to individual needs. As our society is growing older, the consequences of aging have begun to gain particular attention. In particular, skin aging has gained increasing interest, not only because it is the most obvious sign of the aging process but mostly because it represents a window for human health. Skin aging is a complex process in which both intrinsic and extrinsic determinants lead progressively to a loss of structural and morphological characteristics and as a consequence all its functions deteriorate (Zouboulis and Makrantonaki, 2011). To illustrate how aging mechanisms vary depending on their origin, Table 1 compares the clinical and histological features of intrinsic and extrinsic aging across different skin layers, adapted from Farage et al. (2008).

**TABLE 1:** Comparison of Clinical and Structural Characteristics of Intrinsic vs Extrinsic Skin Aging. Adapted from Farage et al. (2008). This table compares key features of intrinsic (chronological) aging and extrinsic (photo-induced) aging in human skin, across different anatomical layers and cellular components.

Skin layer/Feature	Intrinsic aging	Extrinsic aging
Epidermis - Thickness	Thins with aging	Acantosis in early stages
Epidermis - Proliferative Rate	Lower than normal	Higher than normal
Epidermis - Keratinocyte Morphology	Modest cellular irregularity	Atypical polarity
Dermo-epidermal Junction	Modest reduplication of lamina densa	Extensive reduplication of lamina densa
Vitamin A Content	Plasma content of retinol increases	Destroyed by sun exposure
Dermis - Elastin Fibers	Elastogenesis followed by elastolysis (‘moth-eaten’ fibers)	Marked elastogenesis with dense degeneration
Dermis - Elastin Matrix	Gradual decline in matrix production	
Dermis - Lysosome Deposition on Elastic Fibers	Modest increase	Increased significantly
Dermis - Collagen Production	Stable mature collagen, slower degradation	Reduced levels of mature collagen
Grenz Zone	Absent	Prominent
Microvasculature	Microvessels decrease, structure remains unchanged	Dilated, structurally abnormal vessels
Inflammatory Response	None observed	Pronounced perivascular inflammation

Ethnic differences in skin structure and function play a crucial role in shaping skin aging outcomes. Research indicates that up to 60% of skin aging variation can be attributed to genetic factors (Naval et al., 2014), explaining the significant genetic variations among ethnic groups that affect pigmentation, hydration, and barrier integrity (Alexis and Obioha, 2017). For example, African skin exhibits higher melanin levels, contributing to increased photoprotection. *In vitro* studies have also shown that genetic variants, such as those in *MC1R* and *SLC45A2*, may influence pigmentation pathways (Cook et al., 2009), which could help explain the predisposition to post-inflammatory hyperpigmentation observed in clinical settings. Asian skin often displays a thinner stratum corneum and heightened sensitivity to environmental factors, with SNPs in FLG and TYR contributing to barrier dysfunction and pigmentation concerns (Sturm et al., 2003). European skin is often observed to show earlier signs of photoaging, partly due to lower melanin levels. Experimental studies in Sod2-deficient mice have demonstrated that mitochondrial oxidative stress can lead to cellular senescence and skin aging phenotypes, suggesting a potential role for antioxidant defense mechanisms in skin aging (Verlarde et al., 2012).

Comparative studies have identified variations in skin barrier properties, clinical presentations, and responses to environmental stressors among different racial/ethnic groups (Alexis and Obioha, 2017; Alexis et al., 2022). For example, a study evaluating personalized skincare through *in silico* gene interaction networks analyzed the role of single nucleotide polymorphisms (SNPs) in skin responses to ultraviolet radiation (UVR) and oxidative stress across different ethnic groups. The findings demonstrated significant variations in genetic pathways linked to pigmentation, barrier function, and inflammation among European, Asian, and African skin types, reinforcing the potential of genetic profiling to inform tailored skincare formulations (Markiewicz and Idowu, 2022a).

However, while inter-ethnic differences in skin aging are increasingly well documented, intra-ethnic variability—the genetic diversity between individuals within the same ethnic group—remains underexplored. Understanding this dimension is crucial for fully realizing personalized skincare. Even within a single population, individuals may carry SNPs that predispose them to different aging phenotypes. For instance, two individuals of European descent may differ in collagen degradation risk due to variants in MMP1 or COL1A1, or in oxidative stress response due to differences in SOD2 (Minlikeeva et al., 2016). Thus, ethnicity-specific averages can serve as a foundation, but individualized profiles are necessary for precision.

Furthermore, it is important to distinguish between genetic contributors to intrinsic *versus* extrinsic aging. Genes involved in ECM composition (e.g., COL1A1, ELN), telomere maintenance (e.g., TERT), and antioxidant capacity (e.g., SOD2, GPX1) primarily influence intrinsic aging, which reflects the natural biological clock. In contrast, genes regulating pigmentation (e.g., MC1R, TYR, SLC45A2) and inflammatory responses (e.g., IL6, TNF-α) more strongly modulate extrinsic aging by affecting skin responses to UV exposure and environmental stress (Vierkötter et al., 2015).

A recent review by Ng and Chew (2022) expanded this discussion by identifying 44 pleiotropic and hub genes—genes with multiple roles across biological pathways—that are disproportionately associated with skin aging phenotypes. These genes, many of which are also involved in pigmentation, inflammation, and extracellular matrix integrity, may be central regulators of aging. Their findings reinforce the notion that addressing skin aging requires targeting interconnected processes rather than isolated mechanisms. This complexity strengthens the case for multi-pathway, genomically informed skincare strategies.

Despite the growing recognition of ethnic skin differences, there remains a significant lack of diversity in dermatological research and clinical trials (Pandya et al., 2016; Syder and Elbuluk, 2023). For example, skin-of-color content constitutes only 16.3% of dermatology literature, highlighting an underrepresentation of these populations in scientific studies (Wilson et al., 2021). This gap limits the understanding of genetic and phenotypic variations unique to diverse ethnic groups and hinders the development of tailored skincare solutions.

To address this disparity, actionable steps must include prioritizing diversity in clinical trial recruitment and research funding. Initiatives should focus on recruiting participants from underrepresented groups to ensure that findings reflect the needs of all populations. Furthermore, dermatological research should integrate multi-ethnic genome-wide association studies (GWAS) to identify genetic markers relevant to skin-of-color populations. Collaborative efforts between academia, industry, and regulatory bodies can facilitate these goals by creating incentives for inclusive studies and promoting ethical research practices. By broadening representation, the industry can develop more inclusive and effective personalized skincare strategies to meet the needs of diverse populations.

## 4 Biological pathways and genetic variants involved in skin aging

The aging process is governed by interconnected biological pathways that balance repair, regeneration, and damage control. Disruptions in these pathways, influenced by both genetic predispositions and environmental factors, accelerate the visible signs of aging, such as wrinkles, loss of elasticity, and uneven skin tone.

With the increasing accessibility of genotyping services, researchers can now employ systematic approaches such as genome-wide association studies (GWAS), gene expression databases, and genotype-phenotype correlation analyses to better understand the genetic contributions to skin aging.

Genome-Wide Association Studies (GWAS) are research methods that examine the entire genome to identify genetic variations, known as single nucleotide polymorphisms (SNPs), associated with specific traits or conditions, such as skin aging. By analyzing these SNPs, researchers can pinpoint genes and biological pathways that influence how our skin ages. For instance, variations in genes involved in the melanogenesis pathway have been linked to differences in skin pigmentation and the development of age-related features like wrinkles and age spots. Understanding these genetic factors through GWAS provides valuable insights into the complex mechanisms of skin aging, potentially guiding the development of targeted interventions to maintain skin health and appearance.

### 4.1 Melanogenesis pathway

The melanogenesis pathway, primarily known for regulating pigment production in the skin, also plays a significant role in skin aging. Melanin, the pigment responsible for skin color, serves as a natural defense mechanism against ultraviolet (UV) radiation by absorbing and dissipating harmful rays. However, dysregulation of this pathway can contribute to the visible signs of aging, including uneven pigmentation, age spots (solar lentigines), and changes in skin tone uniformity. Chronic UV exposure not only increases melanin production but also leads to oxidative stress and inflammation, compounding the effects of aging on the skin (Brenner and Hearing, 2008).

Melanogenesis is regulated by key genes such as tyrosinase (TYR), tyrosinase-related protein 1 (TYRP1), and dopachrome tautomerase (DCT), which are involved in the enzymatic reactions that produce melanin. Variations in these genes can influence the efficiency and quantity of melanin production. For instance, single nucleotide polymorphisms (SNPs) in TYR, such as rs1126809, have been linked to variations in pigmentation and susceptibility to UV-induced damage (Bastiaens et al., 2001). Another gene, melanocortin 1 receptor (MC1R), plays a central role by modulating the type of melanin produced—pheomelanin (lighter pigment) or eumelanin (darker pigment). Specific variants in MC1R, such as rs1805007, are associated with a reduced ability to produce protective eumelanin, increasing the risk of photoaging and UV-induced damage (Box et al., 2001).

Beyond pigmentation, the melanogenesis pathway interacts with oxidative stress and inflammation pathways. Differences in melanin type and content can influence oxidative stress levels in the skin. Melanocytes with lower eumelanin content produce more reactive oxygen species (ROS) upon UV exposure, which may contribute to cellular damage and skin aging, whereas higher eumelanin levels are associated with better antioxidant protection (Upadhyay et al., 2022). SNPs in the solute carrier family 45 member 2 (SLC45A2) and oculocutaneous albinism II (OCA2) genes, which regulate melanin synthesis and transport, have also been implicated in pigmentation disorders and sensitivity to environmental aging factors like UV radiation (Sturm et al., 2003; Cook et al., 2009).

Furthermore, genes like KIT ligand (KITLG) and endothelin 1 (EDN1), which regulate melanocyte activity and proliferation, contribute to age-related changes in pigmentation patterns. Genetic variants in these genes can alter melanocyte function, leading to uneven pigmentation that is characteristic of aged skin (Imokawa and Ishida, 2015; Yamaguchi and Hearing, 2009). Over time, cumulative UV exposure exacerbates these effects, creating a feedback loop of oxidative stress, inflammation, and pigmentation changes that manifest as visible aging.

### 4.2 Oxidative stress

One critical pathway in skin aging is the oxidative stress pathway, which involves the accumulation of reactive oxygen species (ROS) due to environmental factors like UV radiation and natural metabolic processes (Papaccio, 2022). Over time, ROS damage cellular components such as DNA, proteins, and lipids, contributing to the breakdown of skin structure. The body has antioxidant defense systems to combat ROS, but these mechanisms become less effective with age or due to genetic variations, allowing damage to accumulate (Poljšak et al., 2012).

Research on genetic variations in antioxidant enzymes suggests potential links to aging and disease susceptibility. Studies have found associations between single nucleotide polymorphisms (SNPs) in superoxide dismutase 2 (SOD2), glutathione peroxidase 1 (GPX1), and glutathione S-transferase P1 (GSTP1) genes and various conditions. The SOD2 rs4880 and GPX1 rs1050450 SNPs were associated with longevity and decreased mortality in the oldest old (Soerensen et al., 2009). MnSOD genetic variation was linked to oxidative status, particularly in postmenopausal women (Minlikeeva et al., 2016).

### 4.3 Extracellular matrix

The extracellular matrix (ECM) plays a pivotal role in maintaining the structural integrity and elasticity of the skin. Two key components of the ECM are collagen type I, encoded by the collagen type I alpha 1 chain (COL1A1) gene, and elastin, encoded by the ELN gene. Collagen provides tensile strength to the dermis, while elastin imparts elasticity and resilience to the skin. Single nucleotide polymorphisms (SNPs) within these genes can significantly impact the synthesis and structural organization of these proteins, leading to accelerated skin aging.

The rs1800012 polymorphism in *COL1A1* has been associated with alterations in collagen production and degradation, potentially weakening dermal structure and contributing to signs of skin aging such as wrinkles and reduced firmness (Yilmaz et al., 2023; Nisticò et al., 2018). The rs2071307 polymorphism in *ELN* has been associated with alterations in elastin structure, potentially weakening dermal elasticity and contributing to signs of skin aging such as sagging and reduced firmness (Nisticò et al., 2018). These genetic variations underscore the importance of ECM integrity in the aging process and highlight potential targets for intervention.

Matrix metalloproteinases (MMPs) play a crucial role in skin aging by regulating extracellular matrix (ECM) remodeling and degradation (Freitas-Rodríguez et al., 2017; Fisher et al., 2023). Matrix metalloproteinases (MMPs), particularly MMP-1, play a crucial role in skin aging and photoaging by degrading collagen and elastin in the dermal extracellular matrix (Pittayapruek et al., 2016; Fisher et al., 2009). UV radiation, both UVA and UVB, upregulates MMP activity in skin fibroblasts and keratinocytes, leading to decreased skin elasticity and the formation of wrinkles and sagging (Imokawa and Ishida, 2015). But also genetic variants in matrix metalloproteinase (MMP) genes play a crucial role in skin aging by upregulating MMP activity (Vierkötter et al., 2015).

### 4.4 Telomeres

Another important mechanism involves the telomere maintenance pathway. Telomeres are protective caps at the ends of chromosomes that shorten with each cell division. When they become critically short, cells enter a state of senescence or programmed cell death, impairing the skin’s ability to regenerate and repair damage. Telomere length is therefore a biomarker of aging, with shorter telomeres associated with age-related diseases and early mortality (Blackburn et al., 2015; Rizvi et al., 2015). Genetically determined differences in telomere length affect various biological traits and influence the rate of the ageing process (Codd et al., 2021).

Genome-wide association studies have identified 197 variants at 138 loci associated with telomere length. Notably, SNPs in genes such as telomerase RNA component (TERC), telomerase reverse transcriptase (TERT), and oligonucleotide/oligosaccharide-binding fold containing 1 (OBFC1) have been linked to variations in telomere length. For instance, a study by Codd et al. (2013) identified SNPs in TERC and TERT as significant determinants of telomere length. While these genetic variants influence telomere length, their direct effect on skin aging remains an area of ongoing research. However, a Mendelian randomization study by Zhan and Hägg (2021) found that longer genetically predicted telomeres were associated with a lower likelihood of facial aging, suggesting a potential link between telomere length and skin aging.

Additionally, research has explored the relationship between telomere length and skin cancer. A study published in Frontiers in Genetics by Son et al. (2022) examined the association between telomere length and skin cancer, providing insights into the complex relationship between telomere biology and skin health.

### 4.5 Inflammation

Chronic inflammation, often referred to as inflammaging, is a key contributor to accelerated skin aging. Inflammatory pathways, such as those regulated by NF-κB, are activated in response to cellular stress and injury. This pathway, when persistently activated, can lead to the production of pro-inflammatory cytokines like interleukin-6 (IL-6) and tumor necrosis factor alpha (TNF-α). These cytokines are known to upregulate matrix metalloproteinases (MMPs), particularly MMP-1 and MMP-3, which degrade collagen and elastin in the extracellular matrix (Fisher et al., 2023; Vierkötter et al., 2015). Genetic variations, such as single nucleotide polymorphisms (SNPs) in *IL6* (rs1800795) and *TNF-α* (rs361525), have been associated with heightened inflammatory responses in various diseases, which may indirectly contribute to skin aging through chronic inflammation. Persistent low-grade inflammation also impairs skin repair mechanisms by disrupting fibroblast function and increasing oxidative stress. Together, these processes accelerate extracellular matrix degradation and contribute to the visible signs of aging, including wrinkles, loss of elasticity, and uneven skin texture (Bektas et al., 2018).

### 4.6 Skin barrier and hydration

The skin barrier plays a critical role in maintaining hydration and protecting against environmental insults. Key pathways involved in skin barrier maintenance and hydration include the filaggrin (FLG) and aquaporin (AQP) pathways. Filaggrin, encoded by the FLG gene, is a protein essential for the formation and hydration of the stratum corneum barrier function. Loss-of-function mutations in FLG are the most significant genetic risk factor for atopic dermatitis (AD) and related allergic conditions (Mcaleer and Irvine, 2013; Brown and McLean, 2012). These mutations, present in up to 10% of the population, lead to reduced filaggrin production, resulting in increased transepidermal water loss, skin dryness, and heightened AD risk (Sandilands et al., 2009). FLG mutations are associated with early-onset, persistent eczema, and increased risk of asthma, allergic rhinitis, and multiple allergic sensitizations (Henderson et al., 2008; Weidinger et al., 2008). The prevalence and spectrum of FLG mutations vary between European and Asian populations (Osawa et al., 2011).

Similarly, the aquaporin pathway, regulated by the AQP3 gene, is vital for water transport and hydration within the epidermis (Brandner, 2007; Boury-Jamot et al., 2009). AQP3 facilitates water and glycerol transport, contributing to stratum corneum hydration, barrier recovery, and keratinocyte proliferation (Bollag et al., 2020; Hara-Chikuma and Verkman, 2008). Dysregulation of AQP3 is associated with various skin diseases, including atopic eczema and psoriasis (Liu et al., 2023; Draelos, 2012).

### 4.7 Epigenetics and skin aging

While genetics play a foundational role in skin aging, epigenetic mechanisms significantly modulate gene expression in response to internal and external stimuli. Epigenetics encompasses changes in gene activity that do not involve alterations in the DNA sequence. Key mechanisms include DNA methylation, histone modifications, and the regulation of gene expression by microRNAs (miRNAs). DNA methylation patterns change with age and have been used to create “epigenetic clocks” capable of estimating biological age more accurately than chronological age (Horvath, 2013). Altered methylation of genes associated with collagen production, inflammation, and oxidative stress can contribute to accelerated aging phenotypes. Histone modifications also influence chromatin accessibility, thereby regulating transcriptional activity of aging-related genes. Additionally, miRNAs such as miR-21 have been implicated in modulating extracellular matrix remodelling and inflammatory responses in aging skin (Wang et al., 2019).

### 4.8 Host genetics and the skin microbiome

The skin microbiome—comprising bacteria, fungi, and viruses residing on the skin surface—plays a pivotal role in maintaining skin health, modulating immune responses, and protecting against environmental insults. While microbiome composition is strongly influenced by environmental and lifestyle factors, host genetics also plays a significant role in shaping microbial communities. Recent genome-wide association studies (GWAS) have identified genetic variants associated with the abundance of specific bacterial taxa, including genes involved in immune regulation, epidermal barrier function, and sebum production. For instance, polymorphisms in the FLG gene, which impact barrier integrity, are linked to altered microbial diversity and increased susceptibility to conditions like atopic dermatitis. Variants in genes such as defensin beta 1 (DEFB1) and interleukin-1 (IL-1) family members have also been associated with shifts in microbial populations. As microbial dysbiosis is increasingly recognized as a contributor to skin aging—through chronic inflammation, oxidative stress, and impaired repair—understanding host-microbiome interactions opens new avenues for personalized skincare. Tailoring treatments based on an individual’s genetic and microbiome profile may enhance therapeutic efficacy and support long-term skin resilience.

## 5 Gene–environment interactions and the exposome

While genetic predisposition influences how individuals age, it is the combination of genetic factors with environmental exposures—the so-called exposome—that ultimately shapes skin aging outcomes. The skin exposome includes ultraviolet (UV) radiation, pollution, tobacco smoke, dietary habits, sleep quality, stress, and other external elements that accumulate over time and interact with one’s genetic makeup.

Ultraviolet radiation remains the most significant environmental contributor to extrinsic skin aging. UVB and UVA exposure induce oxidative stress, DNA damage, and upregulation of matrix metalloproteinases (e.g., MMP1), leading to collagen degradation and elastin fiber disorganization. Individuals with certain polymorphisms in genes like MC1R or TYR are more sensitive to UV damage due to lower melanin levels or altered pigment synthesis pathways (Sturm et al., 2003).

Similarly, air pollution and cigarette smoke generate reactive oxygen species (ROS), overwhelming antioxidant defenses. Variants in antioxidant genes such as SOD2 (e.g., rs4880) and GPX1 can further compromise the skin’s ability to neutralize ROS, thereby accelerating wrinkle formation, pigmentation disorders, and inflammation (Vierkötter et al., 2010).

Importantly, environmental stressors can also induce epigenetic modifications—reversible changes in gene expression without altering DNA sequence. These include DNA methylation, histone modification, and microRNA regulation. For instance, UV exposure has been shown to accelerate DNA methylation at promoters of genes involved in inflammation and senescence, such as IL6 and MMPs, leading to phenotypic manifestations of aging even in genetically low-risk individuals (Grönniger et al., 2010).

Thus, the interaction between genetic makeup and environmental exposures determines not just the rate of skin aging, but also its pattern and severity. This highlights the importance of integrating lifestyle assessment into personalized skincare strategies. Individuals carrying risk variants in oxidative stress or barrier genes may benefit more from antioxidant-rich skincare, pollution shields, or barrier-reinforcing agents.

Gene–environment interactions also support a more preventive model of skincare, where early identification of genetic vulnerabilities can inform protective behaviors—such as sun avoidance or antioxidant supplementation—long before clinical signs of aging appear. This approach aligns with the emerging philosophy of predictive and preventive dermatology.

## 6 Polygenic risk scores and predictive dermatology

PRS represent a powerful tool in predictive health, combining the effects of multiple genetic variants across the genome to estimate an individual’s risk for a particular trait or disease. In dermatology, PRS can be used to anticipate predisposition to conditions such as acne, psoriasis, or accelerated aging. For example, a composite PRS incorporating SNPs related to collagen degradation, oxidative stress, and pigmentation could forecast the likelihood of early wrinkle formation or pigmentation irregularities. Despite its potential, the use of PRS in dermatological practice is still in early stages due to the need for large, ethnically diverse GWAS datasets. However, ongoing research is developing PRS models for pigmentation, eczema, and skin cancer that may soon be translated into consumer or clinical applications (Choi et al., 2020).

## 7 Linking genetic profiles with personalised skin care products

A problem inherent in the use of genetic profiling is the huge amount of data produced. Searching for meaningful information patterns and dependencies among genes, in order to provide a basis for hypothesis testing, typically includes the initial step of grouping genes, with similar changes in expression into groups or “clusters.” Clustering is defined as dividing data points or populations into several groups such that similar data points are in the same group. The aim is to segregate groups based on similar traits.

Naval et al. (2014) used the k-means technique for genetic cluster analysis. They confirmed that genetic groups could be linked to biochemical and metabolic skin properties and identified genetic clusters that explained the differences in the effects of the SNP variants on the biochemical and metabolic properties of the skin. This suggests that different skincare needs depend on the naturally occurring genetic variants present in each one of the genetic clusters. This provides the basis for relevant and actionable skincare recommendations that go beyond general advice like using collagen-stimulating products, which are beneficial for most people regardless of genetic results.

The principles of dermagenetics and dermagenomics in personalized skincare focus on addressing genetic variations that affect skin health and the efficacy of topical skincare solutions. Personalized approaches aim to match skincare products to individual genetic profiles, potentially improving efficacy (Markiewicz and Idowu, 2018). Studies have identified SNPs associated with response to specific anti-aging ingredients (Lee and Rong-Mullins, 2020) and developed methods to determine product suitability based on genetic analysis (Toumazou et al., 2012). This precision allows for the formulation of products that address deficiencies caused by genetic variations and ensures that treatments not only alleviate symptoms but also address the root causes of skin concerns, aligning closely with the principles of personalized medicine and enhancing therapeutic outcomes.

Single nucleotide polymorphisms (SNPs) in the FLG gene, such as loss-of-function mutations, result in reduced filaggrin production. This deficiency compromises the skin’s barrier function, leading to increased transepidermal water loss (TEWL) and heightened susceptibility to dryness and irritation. To mitigate these effects, skincare formulations enriched with ‘skin-identical lipids’ like ceramides, fatty acids, and cholesterol can effectively substitute for the weakened barrier components, restoring hydration and reinforcing skin integrity (McAleer and Irvine, 2013).

Genetic variations in SOD2, such as the SNP rs4880, can lead to reduced antioxidant defense mechanisms, impairing the skin’s ability to neutralize reactive oxygen species (ROS). To counteract this deficiency, topical application of SOD mimetics or dietary antioxidant supplementation has shown promise in reducing oxidative injury and tumor incidence in skin cancer models (Robbins and Zhao, 2011). Unlike vitamin C, which must be obtained through diet or topical applications (Drouin et al., 2011), SOD is endogenously produced, making it a suitable biomarker for precision skincare approaches.

Variations in genes like COL1A1 and MMP1 significantly impact collagen production and degradation, which are crucial for maintaining skin firmness and elasticity. Individuals with a genetic predisposition to faster collagen degradation due to overexpression of MMP1 should prioritize treatments that specifically inhibit MMP1 activity, such as those containing targeted peptides or MMP inhibitors. This targeted approach directly mitigates the root cause of collagen breakdown and is best employed in conjunction with collagen-stimulating strategies to achieve comprehensive skin rejuvenation. While peptides and retinoids promote collagen production, these efforts can be undermined if MMP1 activity remains unregulated, as excessive MMP1 levels will continue to degrade newly synthesized collagen. By integrating MMP1 inhibitors alongside collagen-stimulating ingredients, a comprehensive and effective solution can be achieved, ensuring that the benefits of increased collagen synthesis are preserved and fully realized.

Additionally, nutritional supplementation can play a vital role in addressing deficiencies associated with genetic variations (Stover, 2006; Stover, 2007). SNPs have been associated with varying outcomes in collagen supplementation for skin health (Choi et al., 2019). Individuals with these variations may benefit from collagen peptides in combination with vitamin C supplements, which are essential for collagen formation. Genotype-guided supplementation has shown promise in optimizing health benefits and reducing adverse effects (Wang et al., 2022; Peneş et al., 2017), and it becomes increasingly important to consider genetic factors when determining optimal dosages for natural products like collagen supplements (Jhawar et al., 2020; Postlethwaite et al., 2024).

A recent review by Ng and Chew (2022) emphasized that many SNPs associated with skin aging are located within pleiotropic hub genes—such as SOD2, MMP1, and MC1R—that influence multiple biological processes simultaneously. These findings support the need for multi-targeted skincare strategies that address overlapping pathways like oxidative stress, inflammation, and extracellular matrix remodeling.

To bridge the gap between genetic predispositions and practical interventions, Table 2 summarizes key genes and biological pathways associated with skin aging, their functional relevance, associated dermatological risks, and recommended skincare strategies.

**TABLE 2:** Genetic Pathways Involved in Skin Aging and Corresponding Personalized Skincare Strategies. This summary table outlines the functional roles of selected genes and pathways in skin aging, the associated risks or phenotypic outcomes, and targeted skincare strategies that can be used to mitigate these effects in a personalized approach.

Gene/Pathway	Function	Associated risks	Skincare strategy
FLG (Filaggrin)	Maintains skin barrier and hydration	Dryness, increased TEWL, atopic dermatitis	Use ceramide-rich moisturizers; reinforce barrier with fatty acids and cholesterol
AQP3 (Aquaporin 3)	Facilitates water and glycerol transport	Dehydration, oxidative stress sensitivity	Use glycerol-based hydration products; antioxidants to counteract ROS.
COL1A1 (Collagen)	Regulates collagen synthesis	Reduced skin firmness, increased wrinkling	Supplement with peptides, retinoids, and vitamin C to enhance collagen production
MMP1	Degrades collagen and elastin	Accelerated ECM degradation, sagging	Apply MMP inhibitors and targeted peptides to preserve ECM integrity
MC1R	Modulates melanin production	UV sensitivity, hyperpigmentation	Use broad-spectrum sunscreen; tyrosinase inhibitors for pigmentation control
SOD2, GPX1	Neutralize oxidative stress	Cellular damage from ROS	Incorporate antioxidant-rich formulations like coenzyme Q10 or superoxide dismutase
TNF-α, IL-6	Regulate inflammatory response	Chronic inflammation, impaired repair	Use niacinamide and anti-inflammatory ingredients to soothe skin
TERT, TERC	Maintain telomere length	Cellular senescence, reduced regenerative capacity	Explore telomere-supportive formulations and anti-aging peptides

Finally, Geusens et al. (2020) were the first to investigate the efficacy of active ingredients designed to address the specific needs of a certain genetic risk profile. In a randomized, controlled, double-blind split-face study on 25 subjects, precision skincare formulations tailored to genetic risk profiles were compared to a non-personalized comparator product. The results demonstrated that personalized skincare regimens targeting high genetic risk for collagen breakdown and medium risk for low antioxidant production were more effective in reducing wrinkles, improving skin roughness, and protecting against UV-induced oxidative damage. These findings underscore the potential of genetic profiling in advancing personalized skincare solutions, offering scientifically grounded, tailored treatments to address individual needs.

## 8 Multi-omics approaches in precision dermatology

Advancements in systems biology have led to the emergence of multi-omics approaches—integrating genomics, transcriptomics, proteomics, metabolomics, and exposomics—to provide a holistic view of skin health and aging. In dermatology, transcriptomic profiling enables the identification of actively expressed genes during inflammation or UV damage, while proteomics maps the dynamic expression of proteins involved in structural integrity and repair processes. Metabolomics provides insight into biochemical changes that accompany aging, such as oxidative stress markers or lipid profile alterations. Exposomics characterizes the cumulative impact of environmental exposures—including UV radiation, pollutants, and diet—on skin health over time. By integrating these datasets, clinicians and researchers can construct individualized skin health profiles, enabling predictive diagnostics and highly tailored therapeutic strategies (Hasin et al., 2017).

## 9 Systems biology and regenerative dermatology

Systems biology shifts the focus from isolated gene effects to dynamic interactions among molecular pathways. In the context of dermatology, this approach emphasizes how pathways involving oxidative stress, ECM degradation, telomere shortening, and inflammation interact in feedback loops that influence skin aging. For instance, chronic inflammation can exacerbate oxidative damage, which in turn accelerates telomere attrition and extracellular matrix breakdown. Mapping these interactions enables a deeper understanding of the pathophysiology of aging skin and informs regenerative strategies that target multiple pathways simultaneously.

Recent research emphasizes that skin aging is not governed by isolated genetic mutations, but rather by complex network interactions among molecular pathways. Oxidative stress can activate inflammatory responses; chronic inflammation further stimulates matrix metalloproteinases (MMPs), degrading collagen and elastin in the extracellular matrix (ECM). Simultaneously, telomere shortening reduces cellular regenerative capacity, while dysregulated melanogenesis and barrier dysfunction exacerbate visible signs of aging. These interconnected loops reinforce one another, creating a cascade effect that accelerates aging. Network-based modeling of these interactions offers a more accurate framework for understanding the biological aging process and developing multi-targeted, precision interventions.

## 10 Limitations, ethics, and real-world considerations in precision skincare

While the integration of genetic data into skincare holds immense promise, several conceptual, ethical, and practical limitations must be addressed to ensure responsible implementation. Precision skincare is evolving rapidly, but applying genetic testing in real-world contexts—particularly outside of clinical medicine—requires a nuanced understanding of both its capabilities and constraints.

Although genome-wide association studies (GWAS) have identified several single nucleotide polymorphisms (SNPs) associated with skin aging across different populations, it is essential to recognize that genetic variations do not always result in a direct, one-to-one causal relationship. Many SNPs exhibit pleiotropy, meaning a single genetic variant can influence multiple biological processes or phenotypes (Ng and Chew, 2022). For instance, FLG mutations not only affect skin hydration but also increase the risk of allergic conditions like asthma and hay fever, demonstrating their broader systemic impact (Weidinger et al., 2008). This complexity underscores the necessity of interpreting genetic contributions in a broader biological context where multiple pathways interact to shape aging outcomes.

Equally important is the probabilistic nature of genetic information. Having a specific variant associated with a higher risk of collagen degradation or pigmentation irregularities does not guarantee that an individual will develop those concerns—especially if protective environmental or lifestyle factors are in place. Conversely, people without such variations may still exhibit signs of accelerated aging due to poor diet, chronic sun exposure, or stress. Therefore, genetics should be viewed as one piece of the puzzle, to be interpreted in concert with epigenetic, environmental, and behavioral data.

This rationale supports the growing emphasis on the skin interactome—a more comprehensive model that integrates genomic, microbiomic, and exposomic data to reflect the full complexity of skin health and disease (Khmaladze et al., 2020). Personalized skincare strategies that adopt this systems-level approach are likely to be more robust and predictive than those relying on genetics alone.

Ethical considerations must also be addressed, especially regarding data privacy and consumer transparency. Genetic testing generates highly sensitive personal information. Companies offering these services must adhere to rigorous privacy standards, including encryption, secure storage, and clear policies on data use. Consumers should be informed explicitly about how their data will be stored, analyzed, and whether it may be used for research or commercial development. Regulatory frameworks must evolve in parallel to protect consumers from the misuse or unauthorized sharing of genetic data—particularly in non-medical settings where oversight is often limited.

Finally, cost remains a significant barrier to equitable access. Advanced genetic testing and personalized skincare solutions often come with high price tags, potentially widening the gap between those who can afford such innovations and those who cannot. To ensure that the benefits of precision dermatology are accessible to all, it is crucial to invest in cost-effective technologies and develop scalable, affordable models for implementation.

## 11 Conclusion and patient opportunities

The integration of genetic insights into dermatology represents a transformative approach to addressing the complex and diverse needs of patients. By understanding the role of genetic variations in pathways such as collagen synthesis, oxidative stress, melanogenesis, and skin hydration, personalized interventions can target the root causes of dermatological concerns rather than merely addressing symptoms. This shift moves dermatology practices—spanning both skincare and therapeutic interventions—from a generic, one-size-fits-all model to a precise, evidence-based strategy that promises enhanced efficacy and greater patient satisfaction.

Importantly, skin health should be recognized as an essential component of healthy aging. As the skin reflects internal health status and serves as a frontline defense, maintaining its structure and function through personalized, science-based care is a gateway to broader physiological resilience.

Emerging trends in technology are further advancing the potential of personalized dermatology. Artificial intelligence (AI) plays a pivotal role by analyzing vast datasets of genetic information, environmental factors, and clinical metrics to provide tailored recommendations. AI-powered platforms enable the creation of dynamic, evolving treatment plans that adapt to individual needs over time. However, to fully realize the potential of precision dermatology, it is essential to take a broader view of the skin interactome, incorporating insights from the microbiome, epigenetics, transcriptomics, and proteomics.

The microbiome, as a key regulator of skin and systemic health, interacts with genetic and environmental factors to influence inflammation, immunity, and barrier function. Epigenetic modifications, such as DNA methylation and histone changes, provide a dynamic layer of information on how environmental exposures, lifestyle choices, and aging influence gene expression. Transcriptomics and proteomics further deepen our understanding by examining active gene expression and protein pathways, offering insights into the dynamic biological processes underlying skin conditions and aging. Integrating these multi-omics perspectives enables dermatologists to develop holistic interventions that address the intricate interplay of factors influencing skin health and disease.

Personalized approaches in dermatology, informed by this comprehensive understanding, result in more effective solutions for conditions such as acne, eczema, psoriasis, pigmentation disorders, and aging-related concerns. By targeting genetic predispositions, modulating microbiome balance, and accounting for epigenetic influences, these treatments not only address immediate symptoms but also foster long-term resilience and improved skin health. In addition to topical or systemic treatments, patients benefit from lifestyle recommendations tailored to their unique biology, creating a holistic framework for sustained care.

Importantly, personalized dermatology emphasizes inclusivity by considering ethnic, genetic, and environmental diversity, ensuring that interventions are effective for all populations. While initial costs for genetic and multi-omics analyses may be higher, the precision and long-term effectiveness of these interventions reduce wasted expenditures on ineffective treatments, ultimately offering greater value to patients and healthcare systems alike.

To fully embrace precision dermatology, the field must align with the four foundational pillars of precision health:

• Predictive: Leveraging PRS and biomarkers to anticipate dermatological concerns before clinical symptoms manifest.
• Preventive: Using evidence-based interventions—including targeted skincare, nutritional supplementation, and behavioral guidance—to mitigate risks.
• Personalized: Adapting treatments and recommendations based on each individual’s genetic, epigenetic, microbiomic, and exposomic profile.
• Participatory: Empowering patients to engage with their own data and co-design their care using digital tools, direct-to-consumer testing, and clinician-guided insights.

This model not only enhances treatment efficacy but also strengthens the therapeutic relationship between patients and practitioners, encouraging shared decision-making and long-term commitment to skin health.

Nonetheless, challenges remain. The field must prioritize inclusive research, robust validation of biomarkers, and the ethical application of genetic data. Only then can the promise of precision skincare be fully realized—scientifically grounded, accessible, and meaningful across diverse populations.

In conclusion, genetic profiling in dermatology offers more than cosmetic benefit—it opens the door to proactive, equitable, and personalized skin health management as an integral part of healthy aging.
